# Thermodynamics Irreversibilities Analysis of Oxy-Fuel Diffusion Flames: The Effect of Oxygen Concentration

**DOI:** 10.3390/e24020205

**Published:** 2022-01-28

**Authors:** Huibo Yan, Guangtong Tang, Chaoyang Wang, Lujiang Li, Yuanke Zhou, Zhongnong Zhang, Chun Lou

**Affiliations:** 1 State Grid Hebei Energy Technology Service Co., Ltd., Shijiazhuang 050021, China; hbdyyyanb@163.com (H.Y.); tgt114@163.com (G.T.); hbdyywcy@163.com (C.W.); hbdyyllj@163.com (L.L.); 2 Wuhan Jiuzhou 3D Combustion Technology Co., Ltd., Wuhan 430072, China; zhouyuanke@jiuzhou3d.com; 3 State Key Laboratory of Coal Combustion, School of Energy and Power Engineering, Huazhong University of Science and Technology, Wuhan 430074, China; d201980376@hust.edu.cn

**Keywords:** oxy-fuel combustion, entropy generation, thermodynamics irreversibility, hydrocarbon diffusion flame

## Abstract

In studies on the combustion process, thermodynamic analysis can be used to evaluate the irreversibility of the combustion process and improve energy utilization efficiency. In this paper, the combustion process of a laminar oxy-fuel diffusion flame was simulated, and the entropy generation due to the irreversibilities of the radiation process, the heat conduction and heat convection process, the mass diffusion process, and the chemical reaction process was calculated. The effect of the oxygen concentration in the oxidizer on the entropy generation was analyzed. The results indicated that, as the oxygen concentration in the oxidizer increases, the radiative entropy generation first increases and then decreases, and the convective and conductive entropy generation, the mass diffusion entropy generation, the chemical entropy generation, and the total entropy generation gradually increase.

## 1. Introduction

To mitigate the greenhouse effect caused by the emission of carbon dioxide (CO_2_), oxy-fuel combustion technology has been investigated and developed [1,2]. In an oxy-combustion process, where a pure or highly enriched oxygen (O_2_) stream is mixed with recycled flue gas (mainly CO_2_), the nitrogen (N_2_) is replaced by CO_2_, and the oxygen concentration in the flue gas is about 30%, higher than that in air combustion. Due to the significant difference in the chemical and physical properties between CO_2_ and N_2_, the efficiency of oxy-fuel combustion and traditional air combustion is also different, especially when oxygen concentration is enhanced. It is very useful and important to evaluate energy conversion efficiency in terms of thermodynamics second-law analysis, in which entropy generation is a crucial parameter [3]. Som et al. reviewed fundamental investigations on thermodynamics irreversibilities and exergy analysis during combustion processes of various kinds of fuels, and summarized that the local entropy generation in a combustion process is mainly due to the irreversibility of internal heat and mass transfers and to chemical reactions [4].

A series of numerically or experimentally fundamental analyses of entropy generation in lab-scale flames, pilot-scale combustors, and even large-scale furnaces has been recently conducted. Yapıcı et al. calculated local entropy generation rates in laminar methane-air (CH_4_-air) flames and compared the irreversibility of various processes [5]. Chen et al. numerically studied the effects of hydrogen addition on entropy generation in ultra-lean counter-flow CH_4_-air premixed flames [6]. Zhang et al. analyzed the effects of different diluents (argon, N_2_, CO_2_, and N_2_/CO_2_) on exergy losses from hydrogen premixed flames [7]. Besides that, considering the significant role of thermal radiation in high temperature systems, many researchers also analyzed entropy generation due to thermal radiation in flames [8,9,10,11,12]. The research results from Liu et al. [8] on confined jet flames and Makhanlall et al. [9] on diffusion combusting flows demonstrated that radiation entropy generation is also a non-negligible component in thermodynamics second-law analysis in combustion processes. Zhang and Lou et al. [10,11] presented a numerical analysis and experimental investigation of radiation entropy generation in high-temperature systems and large-scale boiler furnaces. Rajabi et al. [12] computationally investigated local and global entropy generation features in gas turbine combustors. Shan et al. [13] calculated and analyzed spectral radiative exergy distribution characteristics in one-dimensional (1-D) furnace cases. Furthermore, the effects of non-gray characteristics of three-atom gases (CO_2_ and H_2_O), soot particles, and boundary walls on radiation entropy generation were evaluated [14,15,16]. Consequently, the relationships between flame temperature, soot formation, local entropy generation, and thermodynamic irreversibility in flames have been quantitatively analyzed [17,18].

Based on the state of the art in the thermodynamics second-law analysis of combustion processes or flames, it can be deduced that an increase in oxygen concentration will change the temperature in flames and sequentially affect the thermodynamic irreversibility and efficiency of the combustion process. However, few researchers pay attention to entropy generation and exergy loss caused by a changing oxygen concentration in flames. Only Shan et al. [19] preliminarily analyzed the radiative exergy and the spectral distribution characteristics of oxy-coal combustion in a 1-D tube furnace under O_2_/CO_2_ atmospheres with different oxygen ratios. It is worth mentioning that investigating laminar diffusion flames based on Computational Fluid Dynamics (CFD) is fundamental in various combustion processes and is necessary for better understanding the thermodynamics irreversibilities of combustion processes.

Therefore, the present work analyzed the effect of oxygen concentration on entropy generation in oxy-fuel flames using CFD codes. The numerical calculation results of laminar coflow diffusion oxy-ethylene flames under different oxygen concentrations in an oxidizer (varying from 30% to 50%) are presented, and a thermodynamics irreversibilities analysis of these diffusion flames is conducted. Some key conclusions are discussed.

## 2. Numerical Methods

### 2.1. Numerical Computation of Coflow Flame

The coflow laminar diffusion flame is widely utilized in fundamental research of numerical combustion computation. In this paper, a code named Coflame [20] combined with a detailed chemical reaction mechanism, soot model, and non-gray radiation model was used to complete the numerical computation. The conservation equations for mass, momentum, energy, and species mass fraction as well as two transport equations of soot particles in a two-dimensional axisymmetric cylindrical coordinate are solved in this code. As with the models in [18], the DLR mechanism [21], the abstraction of H, the C (HACA) soot model [22], the discrete-ordinates method (DOM), and the statistical narrow-band correlated-k model (SNBCK) [23] were included in the Coflame code.

**Figure 1:**
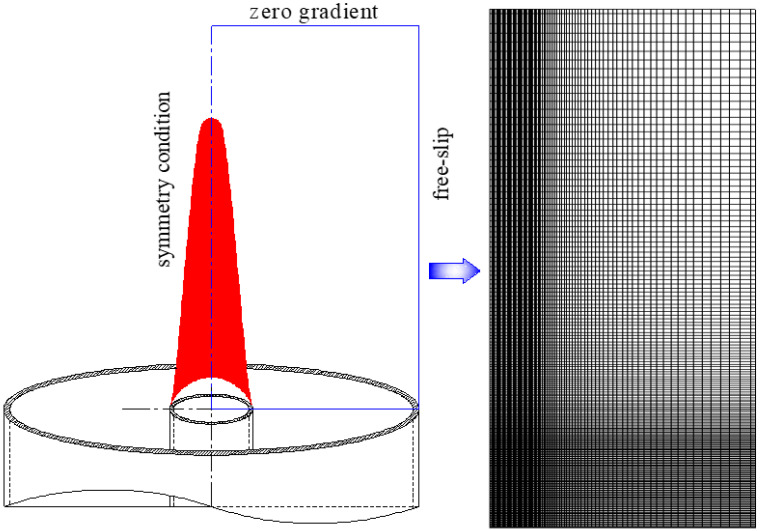
The burner, computation domain, and grid of the coflow laminar diffusion flame.

The burner, flame, and computational domain as well as the grid are shown in Figure 1. Since the coflow laminar diffusion flame is axisymmetric, it can be reduced to a two-dimensional model that contains the axial and radial. As shown in Figure 1, the computational domain is 67 mm (height) × 22 mm (radius) and divided into 164 × 72 control volumes. The grid is non-uniform, and very fine grids (0.24 mm × 0.2 mm) are placed in the main combustion area. The conditions of free-slip and zero-gradient are enforced at the boundary of the coflow stream and exit boundary, respectively. The tip of the burner is considered to be at a constant temperature of 300 K. The computation results that are independent of the grid have been verified in [20].

### 2.2. Calculation of Entropy Generation

According to previous work [3,8,9,18], entropy generation in a flame is mainly due to four irreversible processes: thermal radiation, heat conduction and convection, mass diffusion, and chemical reactions. The total local volumetric entropy generation rate can be computed as follows:

(1) $${S^{\prime \prime \prime }}_{\mathrm{gen}}={S^{\prime \prime \prime }}_{\mathrm{gen},r}+{S^{\prime \prime \prime }}_{\mathrm{gen},\mathrm{cc}}+{S^{\prime \prime \prime }}_{\mathrm{gen},m}+{S^{\prime \prime \prime }}_{\mathrm{gen},\mathrm{ch}},$$

where ${S^{\prime \prime \prime }}_{\mathrm{gen},r}$, ${S^{\prime \prime \prime }}_{\mathrm{gen},\mathrm{cc}}$, ${S^{\prime \prime \prime }}_{\mathrm{gen},m}$, and ${S^{\prime \prime \prime }}_{\mathrm{gen},\mathrm{ch}}$ are the local volumetric entropy generation rates due to thermal radiation, heat conduction and convection, mass diffusion, and chemical reactions, respectively.

Considering the occurrence of three-atom gases (CO_2_ and H_2_O) and soot particles in the hydrocarbon diffusion flame, both of them contribute to the local volumetric entropy generation rate due to thermal radiation and chemistry reactions. ${S^{\prime \prime \prime }}_{\mathrm{gen},r}$, ${S^{\prime \prime \prime }}_{\mathrm{gen},\mathrm{cc}}$, ${S^{\prime \prime \prime }}_{\mathrm{gen},m}$, and ${S^{\prime \prime \prime }}_{\mathrm{gen},\mathrm{ch}}$ can be calculated by Equations (2)–(5), respectively [16,24]:

(2) $${S^{\prime \prime \prime }}_{\mathrm{gen},r}=-\int_{0}^{\infty }\int_{4\pi }\left(\kappa_{\eta ,\mathrm{gas}}+\kappa_{\eta ,\mathrm{soot}}\right)\left[I_{b,\eta }\left(T\right)-I_{\eta }\left(s\right)\right]\left[\frac{1}{T}-\frac{1}{T_{\eta }\left(s\right)}\right]d\Omega d\eta ,$$

(3) $${S^{\prime \prime \prime }}_{\mathrm{gen},\mathrm{cc}}=\frac{k}{T^{2}}\left[{\left(\frac{\partial T}{\partial r}\right)}^{2}+{\left(\frac{\partial T}{\partial z}\right)}^{2}\right],$$

(4) $${S^{\prime \prime \prime }}_{\mathrm{gen},m}=R\rho \sum_{k=1}^{K}\frac{D_{k-\mathrm{mix}}}{X_{k}}\left[\frac{\partial Y_{k}}{\partial r}\frac{\partial X_{k}}{\partial r}+\frac{\partial Y_{k}}{\partial z}\frac{\partial X_{k}}{\partial z}\right],$$

(5) $${S^{\prime \prime \prime }}_{\mathrm{gas},\mathrm{ch}}=-\frac{1}{T}\sum_{n=1}^{N_{1}}\sum_{k=1}^{K}\mu_{k}{\dot{\omega }}_{k,n}$$

where $\kappa_{\eta ,\mathrm{gas}}$ is the absorption coefficient of the gas; $\kappa_{\eta ,\mathrm{soot}}$ is the absorption coefficient of the soot particle; $I_{\eta }$ is the spectral radiative intensity; $T_{\eta }$ is the spectral radiative temperature; k is the conductivity coefficient of the medium; $D_{k-\mathrm{mix}}$ is the mass diffusion coefficient of the *k*th species; $X_{k}$ and $Y_{k}$ are the mole fraction and mass fraction of the *k*th species, respectively; K is the total number of chemical species, $N_{1}$ = 723, and $N_{2}$ = 6 is the total number of reactions in the DLR gas reaction mechanism and the HACA-based soot surface growth oxidation model, ${\dot{\omega }}_{k}$ is the production rate of the *k*th species, $\mu_{k}$ is the chemical potential of the *k*th species, and ω is the net generating rate of the *k*th species.

## 3. Results

The parameters of the working condition calculated in this paper are shown in Table 1. The subscript F represents the fuel, O represents the oxidizer; Q is the volume flow; Y is the volume fraction, χ is the mass fraction. From Case 1 to Case 5, the oxygen concentration in the oxidizer gradually increased from 30% to 50%.

### 3.1. Radiative Entropy Generation

**Figure 2:**
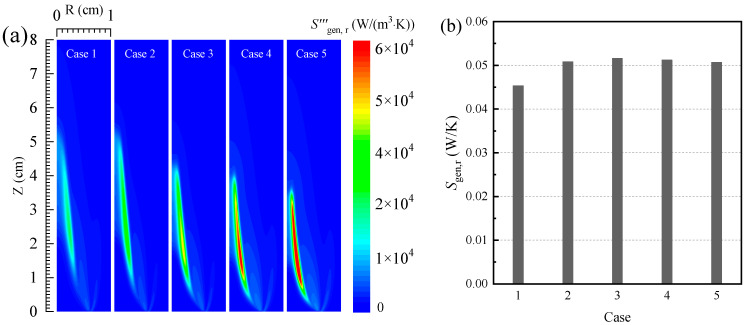
(**a**) The local entropy generation rate and (**b**) the total entropy generation due to the irreversibility of the radiation process.

Figure 2 shows the distribution of the local radiative entropy generation rate and the total radiative entropy generation. It can be seen from Figure 2a that the local radiative entropy generation rate is mainly concentrated on the flame surface. This is because more soot is produced in this area, and the soot particle has an obvious influence on the radiative heat transfer process in the flame. Meanwhile, as the oxygen concentration in the oxidizer increases, the soot volume fraction in the flame gradually increases, the radiative heat transfer process in the flame gradually enhances, and the local radiative entropy generation rate of the flame also gradually increases. For instance, when the oxygen concentration in the oxidizer increases from 30% to 50%, the peak value of the local radiative entropy generation rate in the flame increases from 14,510 to 62,400 W/(m^3^K).

Additionally, according to Figure 2b, it can be seen that, as the oxygen concentration in the oxidizer increases, the radiative entropy generation in the flame presents a trend that first increases and then slightly decreases. When the oxygen concentration in the oxidizer is 40%, the radiative entropy generation of the flame reaches the maximum value (0.05161 W/K). When the oxygen concentration in the oxidizer continues to increase, the volume of the flame gradually decreases, which causes the total radiative entropy generation of the flame to gradually decrease.

### 3.2. Conductive and Convective Entropy Generation

**Figure 3:**
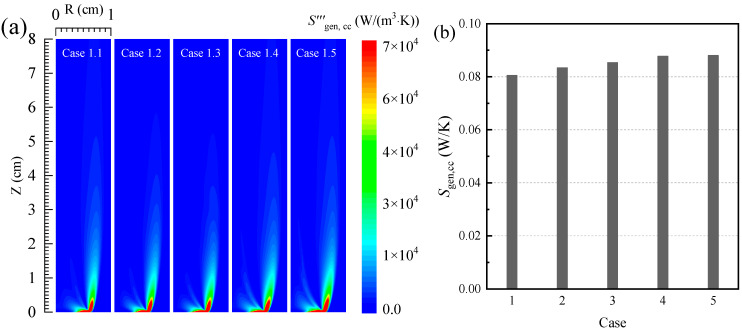
(**a**) The local entropy generation rate and (**b**) the total entropy generation due to the irreversibility of the conductive and convective process.

Figure 3 shows the distributions of the local entropy generation rate as well as the total entropy generation due to heat conduction and convection in the five cases. As shown in Figure 3a, the local entropy generation rate due to heat conduction and convection is mainly concentrated in the region inside and outside the flame surface. Meanwhile, since the temperature gradient of the medium at the tip of the burner is high, the heat conduction and convection process are more intense, and the thermodynamics irreversibilities enhances and local entropy generation rates increase in the region. However, although the temperature of the medium near the flame surface is high, the heat conduction and convection process is weak due to the low temperature gradient of the medium, and the local entropy generation rate due to conduction and convection is low in the region.

Additionally, it can be seen in Figure 3b that, with the increase in the oxygen concentration in the oxidizer, the entropy generation due to heat conduction and convection in the flame increases. When the oxygen concentration in the oxidizer increases from 30% to 50%, the entropy generation due to heat conduction convection in the combustion process increases from 0.081 to 0.088 W/K.

### 3.3. Entropy Generation Due to Mass Diffusion

**Figure 4:**
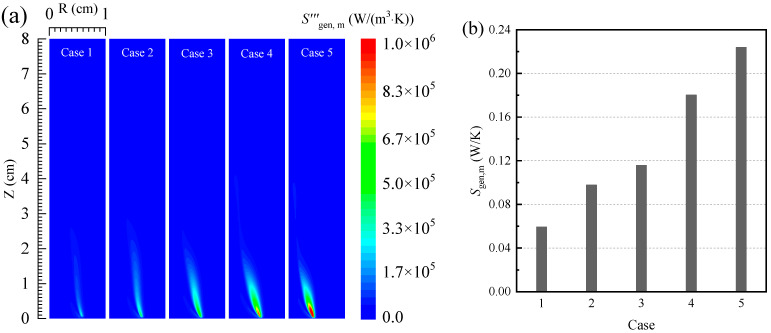
(**a**) The local entropy generation rate and (**b**) the total entropy generation due to the irreversibility of mass transfer.

Figure 4 shows the distributions of the local entropy generation rate as well as the total entropy generation due to mass diffusion in the five cases. As shown in Figure 4a, the local entropy generation rate due to mass diffusion in the flame is mainly concentrated on the flame surface. The local entropy generation rate due to mass diffusion on the tip of the burner significantly increases. The reason is that the concentration gradient of the oxygen is high, and the mass diffusion in the region is intense. Meanwhile, as the oxygen concentration in the oxidizer increases, the concentration gradient of the oxygen near the fuel nozzle gradually increases, and the diffusion process toward the inside of the flame is obviously intensified. The local entropy generation rate due to mass diffusion also gradually increases. Additionally, Figure 4b shows that, with the increase in oxygen concentration in the oxidizer, the total entropy generation due to mass diffusion in the flame increases significantly. When the oxygen concentration in the oxidizer increases from 30% to 50%, the entropy generation due to mass diffusion during the combustion process increases from 0.059 to 0.223 W/K.

### 3.4. Entropy Generation Due to Chemical Reactions

**Figure 5:**
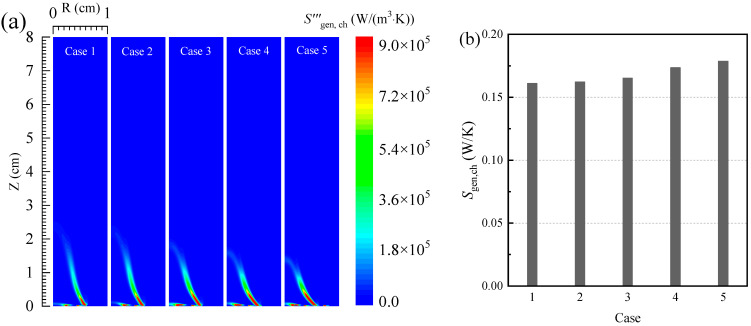
(**a**) The local entropy generation rate and (**b**) the total entropy generation due to the irreversibility of chemical reactions.

Figure 3 shows the distributions of the local entropy generation rate as well as the total entropy generation due to chemical reactions in the five cases. As shown in Figure 5a, the local entropy generation rate due to chemical reactions is concentrated in the region near the nozzle and the region under the flame surface. It is mainly due to the pyrolysis process of the fuel near the flame nozzle and the intense oxidation reaction on the flame surface. Meanwhile, with the increase in oxygen concentration in the oxidizer in the flame, the entropy generation due to chemical reactions in the flame slightly, gradually increases. This indicates that, as the oxygen concentration in the oxidizer increases, the chemical reactions in the flame gradually strengthen. When the oxygen concentration in the oxidizer increases from 30% to 50%, entropy generation due to chemical reactions in the combustion process increases from 0.161 to 0.178 W/K.

**Figure 6:**
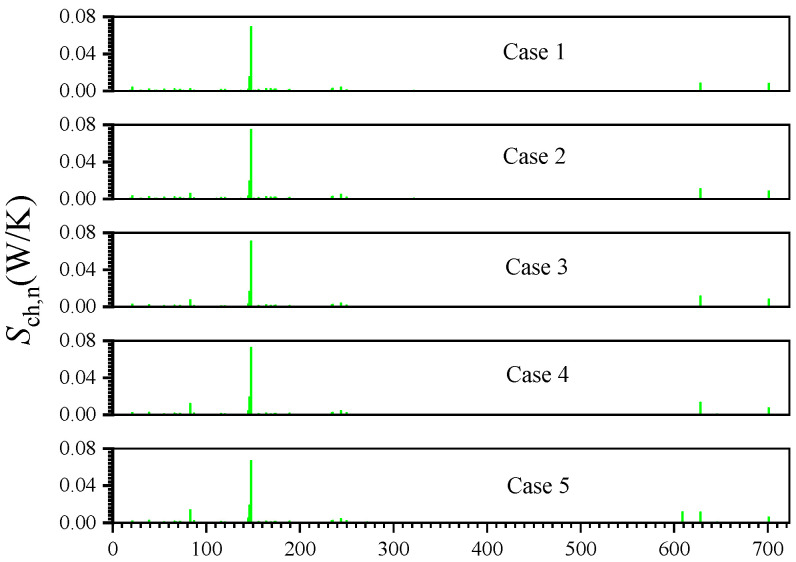
Entropy generation due to the irreversibility of various chemical reactions.

Figure 6 shows the chemical entropy generation by each chemical reaction $S_{\mathrm{ch},n}$ ($S_{\mathrm{ch},n}=-\int_{V}\frac{1}{T}\sum_{k=1}^{K}\mu_{k}{\dot{\omega }}_{k,n}dV$). It can be seen that the reaction R148 has a significant impact on the thermodynamics irreversibilities of the chemical reaction, and the chemical entropy generation due to the reaction exceeds 50% of the total chemical entropy generation. Additionally, there are five other reactions that also have a high impact on chemical entropy generation, and the detailed chemical formula is shown in Formula (6).

(6) $$\begin{array}{l} R83:C+CH_{2}=C_{2}H+H\\ R145:2H_{2}OH+M=CH_{2}O+H+M\\ R146:2HCCO=C_{2}H_{2}+H\\ R148:OH+H_{2}=H_{2}O+H\\ R628:A1C_{2}H+A1C_{2}H=A_{4}+H\\ R700:BAPYR+OH=CH_{2}CO+C_{18}H_{11} \end{array}$$

### 3.5. Total Entropy Generation

**Figure 7:**
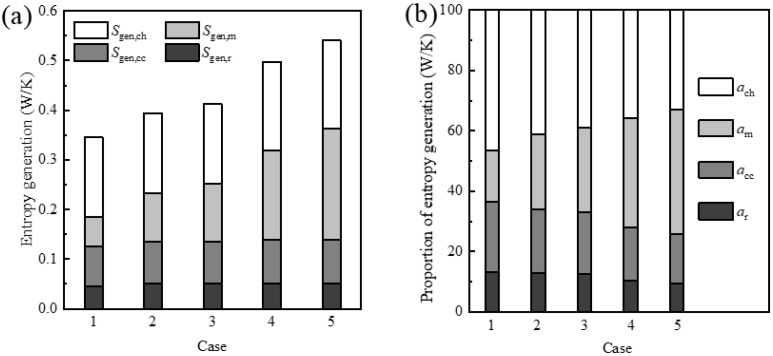
(**a**) Total entropy generation in the combustion process and (**b**) the proportion of entropy generation due to the irreversibility of various processes.

Figure 7 shows the total entropy generation in the combustion process and the proportion of entropy generation due to the irreversibility of various processes. The total entropy generation increases as the oxygen concentration in the oxidizer increases. Meanwhile, as the oxygen concentration in the oxidizer gradually increases, the proportion of entropy generation due to chemical reactions and heat transfer processes gradually decreases. However, the proportion of entropy generation due to the mass diffusion process gradually increases with the increase in the oxygen concentration in the oxidizer.

## 4. Conclusions

In this paper, the entropy generation in the oxy-flame is numerically calculated, and the influence of the oxygen concentration in the oxidizer on the entropy generation in the combustion process is analyzed. The main conclusions are given as follows. Firstly, the local radiative entropy generation rate, the local mass diffusion entropy generation rate, and the local chemical entropy generation rate are mainly concentrated near the flame surface, while the conductive and convective entropy generation is concentrated in the regions inside and outside the flame surface. Secondly, the chemical entropy generation during the combustion process is mainly due to the reaction OH + H_2_ = H_2_O + H, and the entropy generation due to this reaction reaches more than 50% of the total entropy generation. Finally, as the oxygen concentration in the oxidizer increases, the radiative entropy generation first increases and then slightly decreases. The heat conductive and convective entropy generation, the mass diffusion entropy generation, the chemical entropy generation, and the total entropy generation increase.

## Figures and Tables

**Table 1:** Description of 5 flame cases.

Case	$Q_{{F,C}_{2}H_{4}}\;(\mathrm{mL}/\min)$	$Y_{{O,O}_{2}}\;(/)$	$\chi_{O,O}{}_{{}_{2}}\;(\%)$	$Q_{O,O}{}_{{}_{2}}\;(L/\min)$	$Q_{O,\mathrm{CO}}{}_{{}_{2}}\;(L/\min)$
1	194	0.238	30	85.2	198.8
2	194	0.281	35	99.4	184.6
3	194	0.327	40	113.6	170.4
4	194	0.373	45	127.8	156.2
5	194	0.421	50	142.0	142.0

## Data Availability

All data included in this study are available upon request by contact with the corresponding author.
